# Comparison of effective impact among tonsillectomy alone, tonsillectomy combined with oral steroid and with steroid pulse therapy on long-term outcome of immunoglobulin A nephropathy

**DOI:** 10.1007/s10157-012-0679-2

**Published:** 2012-08-29

**Authors:** Yoshihiro Yamamoto, Yoshiyuki Hiki, Shigeru Nakai, Koichiro Yamamoto, Kazuo Takahashi, Shigehisa Koide, Kazutaka Murakami, Makoto Tomita, Midori Hasegawa, Shiro Kawashima, Satoshi Sugiyama, Yukio Yuzawa

**Affiliations:** 1 Department of Nephrology, Fujita Health University Hospital, 1-98 Dengakugakubo Kutsukake-cho, Toyoake, Aichi 470-1192 Japan; 2 Fujita Health University Hospital, School of Health Science, 1-98 Dengakugakubo, Kutsukake-cho, Toyoake, Aichi 470-1192 Japan; 3 Tono Kosei Hospital, Mizunami, Japan; 4 Kanayama Clinic, Nagoya, Aichi Japan

**Keywords:** IgA nephropathy, Tonsillectomy, Steroid therapy, Treatment

## Abstract

**Background:**

To clarify the therapeutic impact of tonsillectomy and combined therapies of tonsillectomy plus steroid on the long-term prognosis of immunoglobulin A nephropathy (IgAN).

**Methods:**

A retrospective study was conducted on 208 patients with IgAN between 1986 and 2009. According to the strategies for treatments, patients were divided into four groups: tonsillectomy and steroid pulse (TSP, *n* = 47), tonsillectomy and oral steroid (TOS, *n* = 33), tonsillectomy alone (T, *n* = 56), and N group (no particular therapy, *n* = 72). Multivariate analysis based on the Cox’s regression model was used to assess the relative risk of reaching the outcome of doubling creatinine based on the influence of baseline prognostic factors.

**Results:**

The mean observation periods were 53.8 months in the TSP group, 122.0 months in the TOS group, 102.9 months in the T group, and 84.6 months in the N group. During an observation period, serum creatinine levels doubled as follows: one in the TSP group (2.1 %), two in the TOS group (6.1 %), five in the T group (8.9 %), histological severity, and 22 in the N group (30.6 %). The Cox’s regression proportional hazard model showed that gender, age, histological activity, dialysis induction risk and therapy were associated with doubling creatinine levels. Hazard ratios (95 % CI) and (*P* value) in T, TOS, and TSP groups versus N were 0.314 (0.11–0.93, *P* = 0.037), 0.213 (0.04–1.10, *P* = 0.065), and 0.032 (0.00–0.28, *P* = 0.002), respectively.

**Conclusion:**

A combination therapy of tonsillectomy and steroid pulse had the most significant therapeutic impact compared to other therapies.

## Introduction

Immunoglobulin A nephropathy (IgAN), characterized by the predominant deposition of IgA in the mesangium, is the most frequent primary glomerulonephritis worldwide as well as constituting ≥ 30 % of adult chronic glomerulonephritis in Japan [1, 2]. The slow progression to end-stage renal disease is known to occur in up to 30–40 % of patients within 20 years [3]. However, a variety of clinical and pathological features emerge while its prognosis varies greatly from case to case. An effective therapeutic modality remains to be established despite the great number of therapeutic trials that have been tried [4–6]. Therefore, we considered it necessary to establish a therapeutic strategy taking into account gender, age, histological findings, and laboratory characteristics.

Regarding the treatment of IgAN, Xie et al. [7] reported on the efficacy of tonsillectomy in 2003. On the other hand, Pozzi et al. [8, 9] reported the effectiveness of steroid pulse therapy based on a series of randomized control trials in 1999 and 2004. Tonsillectomy plus steroid pulse therapy has rapidly spread in Japan. Recently, Kawamura et al. [10] proposed the domestic clinical guidelines for IgAN in Japan, v. 3 (referred to hereafter as CGJ-IgAN) in which dialysis induction risk groups were stratified by prognostic grades that took into account histological as well as clinical severities (Tables 1, 2, 3).

**Table 1:** Classification of clinical severity of IgAN

Clinical severity	Urinary protein (g/day)	eGFR (ml/min/1.73 m^2^)
C-grade I	<0.5	–
C-grade II	≥0.5	≥60
C-grade III	≥0.5	<60

*eGFR* estimated glomerular filtration rate (ml/min/1.73 m^2^)

**Table 2:** Classification of pathologic severity of IgAN

Pathologic severity	Number of glomeruli with global sclerosis + segmental lesion/total number of glomeruli (%)	Acute lesions only	Acute + chronic lesions	Chronic lesions only
H-grade I	0–24.9	A	A/C	C
H-grade II	25–49.9	A	A/C	C
H-grade III	50–74.9	A	A/C	C
H-grade IV	≥75	A	A/C	C

Acute lesion (A): cellular crescent, fibrocellular crescent, glomerular capillary necrosis, chronic lesion (C): nodular sclerosis, segmental glomerulosclerosis, fibrous crescent, segmental lesion: cellular crescent, fibrocellular crescent, segmental sclerosis, fibrous crescent

**Table 3:** Dialysis induction risk

	H-grade I	H-grade II	H-grade III
C-grade I	Low	Moderate	High
C-grade II	Moderate	Moderate	High
C-grade III	High	High	Very high

Proper therapeutic options for IgAN cannot be provided unless pathological diagnosis can be standardized as reliable prognostic indicators. Although such therapeutic options have not been tried, especially with angiotensin-converting enzyme inhibitors (ACEIs) or angiotensin receptor blockers (ARBs), tonsillectomy, tonsillectomy plus oral steroids, and tonsillectomy plus steroid pulse therapy, a comparative evaluation of the long-term outcome of these therapies has not yet been performed.

This study examined the efficacy of several factors impacting long-term renal survival, such as gender, age, therapeutic option, and dialysis induction risk according to the new domestic CGJ-IgAN. Multivariate analysis was used for this study.

## Materials and methods

### Patients

**Fig. 1:**
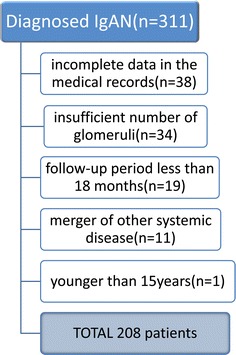
Enrollment of study patients. Detailed list of reasons for exclusion of patients

Between December 1986 and July 2009, 303 patients were diagnosed with IgAN by renal biopsy at Fujita Health University and its affiliated hospitals. The diagnosis of IgAN was based on predominant mesangial IgA staining shown on immunofluorescence study. Patients with systemic diseases such as diabetes mellitus, systemic lupus erythematosus, abnormal hypergammaglobulinemia, chronic liver diseases, and Henoch-Schönlein purpura were distinguished from IgAN by clinico-pathological features. Among IgAN patients, the following patients were excluded from this study: (1) age <15 years, (2) insufficient number of glomeruli (<7 glomeruli) in a biopsy specimen for light microscopic study, (3) follow-up period <18 months, (4) patients who showed a combination with other systemic diseases (antineutrophil cytoplasmic antibodies-associated vasculitis, systemic lupus erythematosus, malignancy) during an observation period, or (5) incomplete data in the medical records. As a result, 208 of the 303 patients were included in this study (Fig. 1).

This study complied with the Helsinki declaration and was approved by the Ethics Committee of Fujita Health University (approval number 11–130).

### Clinical, laboratory, and pathological analyses

The baseline data at the time of renal biopsy were compiled from medical records. The time of renal biopsy was regarded as the entry time into the follow-up. The clinical data evaluated included gender, age, and receiving ACEIs or ARBs. The laboratory data were also evaluated, and included serum creatinine, estimated glomerular filtration rate (eGFR), and degrees of proteinuria and hematuria at (a) the time of renal biopsy, (b) the end of steroid pulse therapy, (c) the end of administration of prednisolone, and (d) the final observation time. The qualitative findings of hematuria were converted into scores as (−) to 0, (±) to 1, (1+) to 2, (2+) to 3, and (3+) to 4. The histological findings were classified according to the new histological classification of IgAN in CGJ-IgAN. The classification details are shown in Tables 1, 2, 3. The names of the patients were blinded to all evaluations of baseline data from renal biopsies.

### Stratification of dialysis induction risk

Predictive grading of dialysis induction risk in the CGJ-IgAN was defined by stratification of the two grades of clinical and histological severities. The clinical severities were graded by the levels of urinary protein (UP g/day) and eGFR (ml/min/1.73 m^2^) at the time of renal biopsy. Clinical grades (C-G I–III) were defined as C-G I, UP < 0.5; C-G II, UP ≥0.5 and eGFR ≥60; C-G III, UP ≥0.5 and eGFR < 60 (Table 1). The parameters of histological evaluation consisted of crescent formation and segmental/global glomerular sclerosis. Thus, histological severity was evaluated by the percentage of injured glomeruli in the total number of glomeruli seen in renal biopsy. Histological grades (H-G I–IV) were defined as H-G I, <25 %; H-G II, 25–49.9 %; H-G III, 50–74.9 %; and H-G IV, ≥75 % (Table 2). Cellular and fibrocellular crescents were defined as acute lesions. Global/segmental glomerulosclerosis or fibrous crescents were defined as chronic lesions. From the clinical and histological grading, dialysis induction risks were stratified and classified as low, moderate, high and very high risk groups as shown in Table 3.

### Treatment protocol

The 208 patients in this study were divided into 4 groups based on the treatment regimens as follows: (1) tonsillectomy alone (T group), (2) tonsillectomy followed by 40 mg/day of oral prednisolone (PSL) which was gradually tapered over 2 years (TOS group), (3) tonsillectomy plus steroid pulse of intravenous methylprednisolone 500 mg/day for 3 consecutive days, generally for 4 courses every 2 months which was discontinued at 3 courses if urinary findings showed remission, followed by oral PSL at an initial dose of 20 mg/day (TSP group), and (4) no particular therapy, in which patients received neither tonsillectomy nor steroid therapy (N group). All patients were given an antiplatelet agent, antithrombotic drugs, and antihypertensive agents according to the discretion of the physician. Among all groups, the use of ACEIs or ARBs was defined as >6 months.

### Statistical analysis

The endpoint of renal survival was set as doubled creatinine levels compared with values at the time of renal biopsy. Cox’s proportional hazards model was used to explore the multiple covariates for renal survival. All continuous variables are presented as mean ± SD. Baseline clinical data among the groups were compared using the Kruskal–Wallis test, unpaired *t* test, and Mann–Whitney *U* test as appropriate for continuous data, and the Chi-squared statistic for categorical data. Cox’s regression proportional hazards model was used to estimate the relative risks associated with the baseline covariates of gender, age, histological activity, the dialysis induction risk, therapeutics, and the use of ACEIs or ARBs. A backward stepwise method was used to select the significant covariates. *P* < 0.05 was used to reject the null hypothesis of no statistical difference between-groups. For the comparison of four groups, Dunn’s test was performed. A *P* value < 0.0083 was considered statistically significant, as indicated by asterisks in the tables. All of the analyses were made using SPSS statistical software for Windows, release Ver.18.

## Results

### Study population

The clinical features of the patients are shown in Table 4. The mean duration of follow up was 88.5 months in the 208 patients (median 76 months, range 18–288 months). The mean age was 35.1 years. The male:female ratio was 1:1. At the time of renal biopsy, mean proteinuria was 1.32 ± 1.50 (SD) g/day. Severe proteinuria (>3.5 g/day) was observed in 10 patients (4.8 %). During the follow-up period, 154 (74.0 %) of the 208 patients were given ACEIs or ARBs. Among the four therapy groups, there were significant differences in eGFR (*P* = 0.001), proteinuria (*P* < 0.001), the dialysis induction risk (*P* = 0.001), and observation period (*P* < 0.001). No difference was observed in the sex ratio, age, hematuria, and use of ACEIs or ARBs. eGFR was significantly lower in the TSP group than in the T and TOS groups. Proteinuria was significantly higher in both TOS and TSP groups than in the T and N groups. The distribution of patients for dialysis induction risk was significantly different among the four groups, with the T and TOS groups having more patients with low risk than the TSP groups.

**Table 4:** (a) Baseline characteristics and (b) distribution of 208 patients with IgA nephropathy

	T group	TOS group	TSP group	N group	*P* value	Total
(a)						
Number of patients	56	33	47	72		208
Sex (male/female)	27/29	17/16	20/27	40/32	0.568	104/104
Age (years)	32.7 ± 13.5	31.4 ± 11.1	34.4 ± 11.0	39.1 ± 15.3	0.250	35.1 ± 13.7
Serum creatinine (mg/dl)	0.85 ± 0.30	0.80 ± 0.20	1.03 ± 0.43	1.04 ± 0.55	0.380	0.95 ± 0.43
eGFR (ml/min)	84.4 ± 27.5	86.5 ± 24.1	67.8 ± 26.7*	72.0 ± 32.3	0.001	76.7 ± 29.6
Proteinuria (g/day)	1.05 ± 1.35	1.71 ± 1.46**	1.87 ± 2.12**	0.98 ± 0.86	<0.001	1.32 ± 1.50
Hematuria	3.4 ± 1.1	3.7 ± 0.6	3.4 ± 1.0	3.2 ± 1.1	0.373	3.4 ± 1.0
Dialysis induction risk (low:moderate:high:very high)	17:29:5:5	3:27:2:1	5:19:9:14*	18:23:17:14	0.001	43:98:33:34
Hypertension (yes/no)	75.0 (42/14)	81.8 (27/6)	78.7 (37/10)	79.2 (57/15)	0.888	74.0 (163/45)
Use of ACEIs or ARBs (%) (use/no use)	69.6 (39/17)	78.8 (26/7)	76.6 (36/11)	73.6 (53/19)	0.774	74.0 (154/54)
Observation period (months)	102.9 ± 51.4	122.0 ± 50.0	53.8 ± 38.1***	84.6 ± 56.8	<0.001	88.5 ± 55.3
Median (months)	100 (24–288)	108 (40–208)	42 (18–204)***	66 (18–258)****		76 (18–288)
(b)						
Histological grade (I:II:III:IV)	40:10:4:2	24:8:1:0	15:14:11:7*	37:15:11:9	<0.001	116:47:27:18
Clinical grade (I:II:III)	18:29:9	3:27:3	8:20:19^†^	22:25:25	0.048	51:101:56
Histological activity (A:A/C:C)	5:10:41^‡^	5:16:12	2:36:9	2:21:49^‡^	<0.001	14:83:111

Data shown as mean ± SD or frequencies. Hematuria were converted into scores as (−) to 0, (±) to 1, (1 +) to 2, (2 +) to 3, and (3 +) to 4. N group patients received neither tonsillectomy nor steroid therapy

*eGFR* estimated glomerular filtration rate (ml/min/1.73 m^2^), *ACEI* angiotensin-converting enzyme inhibitor, *ARB* angiotensin-II receptor blocker, *T group* tonsillectomy alone, *TOS group* tonsillectomy + oral PSL, *TSP group* tonsillectomy + steroid pulse, *N group* no particular therapy

* Significantly different versus T and TOS groups, ** significantly different versus T and N groups, *** significantly different versus T, TOS, and N groups, **** significantly different versus TOS group, ^†^ significantly different versus T group, ^‡^ significantly different versus TOS and TSP groups

### Clinical outcomes

At the final observation, 5 (8.9 %) patients in the T group, 2 patients (6.1 %) in the TOS group, 1 patient (2.1 %) in the TSP group, and 22 patients (30.6 %) in the N group had reached the endpoint of a doubled creatinine concentration since the time of renal biopsy (Table 5). Table 6 shows the eGFRs and urinary protein levels at the times of renal biopsy and at the final observation in each of the 4 groups. The levels of eGFR were significantly decreased in T, TOS, and N groups but not in the TSP group. Except for the N group, urinary protein levels were significantly improved at the final observation. Especially in the steroid therapy groups (TOS and TSP) the average daily urinary protein excretion decreased from >1.5 to <0.5 g/day.

**Table 5:** Outcome of treatment in the each group

	Doubling serum creatinine (%)
T group	5/56 (8.9)
TOS group	2/33 (6.1)
TSP group	1/47 (2.1)
N group	22/72 (30.6)

*PSL* prednisolone, *T group* tonsillectomy alone, *TOS group* tonsillectomy + oral PSL, *TSP group* tonsillectomy + steroid pulse, *N group* no particular therapy

**Table 6:** (a) eGFR and (b) proteinuria in each group

	At renal biopsy	Final observation	*P* value
(a) eGFR (ml/min)			
T group	84.4 ± 27.5	72.5 ± 29.6	<0.001
TOS group	86.5 ± 24.1	77.3 ± 27.6	0.006
TSP group	67.8 ± 26.7	67.7 ± 26.0	ns
N group	72.0 ± 32.3	54.5 ± 38.0	<0.001
(b) Proteinuria (g/day)			
T group	1.05 ± 1.35	0.49 ± 1.16	<0.001
TOS group	1.71 ± 1.46	0.25 ± 0.33	<0.001
TSP group	1.87 ± 2.12	0.42 ± 0.80	<0.001
N group	0.98 ± 0.86	1.07 ± 1.65	ns

*eGFR* estimated glomerular filtration rate (ml/min/1.73 m^2^), *ns* no significant difference, *T group* tonsillectomy alone, *TOS group* tonsillectomy + oral PSL, *TSP group* tonsillectomy + steroid pulse, *N group* no particular therapy

### Risk factors for the development of renal failure

Multivariate hazard ratios for the doubling of serum creatinine levels are shown in Table 7(a). Both gender (male) and age (>40 years) were significant factors in the development of renal failure (*P* < 0.05 for both). Conversely, there was no difference in whether or not ACEIs or ARBs were used. The hazard ratio (HR) for the doubling of serum creatinine levels in histologically judged acute + chronic lesions was 2.53 (95 % CI 1.03–6.17) (*P* < 0.05) and significantly higher than chronic lesions alone. On the other hand, histological findings of acute lesions did not affect the risk of doubling serum creatinine levels. For analysis of the efficacy of the dialysis induction risk, we conducted univariate analysis about each parameter (eGFR, urinary protein, histological grade). eGFR, urinary protein and histological grade were significant factors in the development of renal failure [Table 7(b)]. In the patients in the very high dialysis induction risk group the HR of doubling the serum creatinine level was 12.50, which was also significantly higher than the moderate risk group (*P* < 0.01). The low and high dialysis induction risk patients showed no difference to the moderate risk patients. As for the therapeutics, the HR of the T and TSP groups were 0.314 (0.11–0.93) and 0.032 (0.00–0.28), respectively, compared to the N group (*P* < 0.05, < 0.01). The HR for doubling serum creatinine levels of the TOS group showed no difference with the N group [HR 0.213 (0.04–1.10), *P* = 0.065].

**Table 7:** (a) Multivariate-adjusted and (b) univariate hazard ratios for development of 100 % increase of serum creatinine

	*B*	Standard error	Wald	*P* value	HR	95 % CI
(a)						
Male (vs. female)	1.013	0.459	4.876	0.027	2.76	1.22–6.77
Age (vs. ≤40 years)	1.075	0.419	6.577	0.010	2.93	1.29–6.66
Histological activity (chronic)					1 (reference)	
Acute	−10.023	429.684	0.001	0.981	<0.001	0.00– <1000
Acute + chronic	0.926	0.456	4.123	0.042	2.53	1.03–6.17
Dialysis induction risk (moderate)					1 (reference)	
Low risk	−11.481	205.756	0.003	0.956	<0.001	–
High risk	1.003	0.587	2.916	0.088	2.73	0.86–8.61
Very high risk	2.526	0.540	21.860	0.000	12.50	4.34–36.0
Method of therapy (N)					1 (reference)	
T group	−1.159	0.554	4.372	0.037	0.314	0.11–0.93
TOS group	−1.545	0.837	3.410	0.065	0.213	0.04–1.10
TSP group	−3.449	1.114	9.588	0.002	0.032	0.00–0.28
Use of ACEI or ARB (vs use)	0.956	0.522	3.355	0.067	2.60	0.94–7.24
(b)						
eGFR > 60 ml/min/1.73 m^2^					1 (reference)	
<60 ml/min/1.73 m^2^	1.992	0.405	24.206	<0.000	7.33	3.31–16.2
Urinary protein < 0.5 g/day					1 (reference)	
>0.5 g/day	2.227	1.029	4.686	0.030	9.29	1.23–69.7
Histological grade (I)					1 (reference)	
II	1.424	0.588	5.870	0.015	4.16	1.31–13.2
III	2.031	0.561	13.127	<0.000	7.62	2.54–22.9
IV	2.916	0.563	26.851	<0.000	18.47	6.13–66.7

*PSL* prednisolone, *TSP group* tonsillectomy + steroid pulse, *N* no particular therapy, *T* tonsillectomy alone, *TOS group* tonsillectomy + oral PSL, *ACEI* angiotensin-converting enzyme inhibitor, *ARB* angiotensin-II receptor blocker, *eGFR* estimated glomerular filtration rate (ml/min/1.73 m^2^)

### Adverse effect

Three patients developed steroid-induced psychosis (one in TOS group, two in TSP group). Three patients developed diabetes mellitus and required insulin (one in TOS group, two in TSP group) and received treatment. One patient in the N group died of pneumonia before the endpoint. No patient had any serious side-effect such as aseptic necrosis of femoral bone.

## Discussion

The purpose of this study was to clarify effects of each treatment method on long-term renal survival in adult IgAN patients. To our knowledge, there is no report available from a single institution that compares long-term renal survival among the above treatment methods in adult patients with IgAN. In our institution, tonsillectomy has been performed for patients with IgAN for 25 years. In our institute, TSP therapy was started in 2003. Before 2002, there were no definite criteria of the selection of the treatments (T, TOS, and N). From 2003, we informed the patients of treatment with N, T, and TSP. We recommended TSP to patients if they had urinary protein > 0.5 g/day continuously. However, we also accepted the desire of patients who wished to receive TSP or tonsillectomy. Treatment methods have been applied to cases of various degrees of severity, providing us with a sufficient number of study patients. We employed the technique of multivariate analysis to assess the impact of multiple covariates for long-term renal survival (and to exclude potential bias).

Gender (male), age (≥40 years), histologically acute + chronic region, dialysis induction risk, and therapeutic option significantly affected renal survival. Conversely, use of ACEIs or ARBs did not influence renal survival. A noteworthy result of our study was that tonsillectomy alone significantly contributed to preservation of renal function. This was proved by comparing the T and N groups, both of which did not have a significant difference in clinical and laboratory data (Table 4).

Regarding steroid therapy for IgAN, Kobayashi et al. [11] first reported on its efficacy. Hotta et al. reported the absence of progressive renal dysfunction in 157 IgAN patients that went into so-called ‘clinical remission’ out of 529 patients. Furthermore, they were free of urinary findings after follow-up of ≥ 36 months (average follow-up 82.3 months). Remission was significantly correlated with tonsillectomy and steroid pulse therapy, indicating that it was a potential standard therapy to induce clinical remission [12]. Recently long-term follow-up studies conducted over 10 years were reported concerning the efficacy of tonsillectomy in IgA nephropathy. Akagi et al. [13] and Xie et al. [4] reported that the tonsillectomy group ‘preserved renal function’ more efficiently than in the non-tonsillectomy group. In Japan where health checkup systems are quite advanced, it is relatively easy to detect early-stage IgAN. Therapeutic interventions such as tonsillectomy, when initiated in early-stage IgAN, are expected to preserve the kidney for a longer period. Moreover, our results showing the inhibitory effect of tonsillectomy on progress of IgAN supports the idea that tonsillectomy alone significantly prolonged survival time of the kidney.

According to Katafuchi et al. [14] steroid pulse therapy significantly inhibited the progress of IgAN to terminal renal failure as compared to both non-steroid and oral steroid therapies. These observations were supported by our current study. TSP had the highest impact on inhibiting progression of IgAN. From this observation, it was suggested that tonsillectomy plus steroid pulse therapy was an efficacious therapy to preserve renal function. However, the data of our study provided limited information because this was a retrospective study.

In conclusion, combination therapies of tonsillectomy and steroid pulse had the most significant therapeutic impact compared to other therapies. Multivariable analysis also showed that gender, age, histological activity and the dialysis induction risk were associated with the progression of IgAN.

## References

[1] D‘Amico G. The commonest glomerulonephrites in the world. IgA nephropathy. Q J Med. 1987;64:709–27. 3329736

[2] Levy M, Berger J (1988). Worldwide prospective of IgA nephropathy. Am J Kidney Dis.

[3] Koyama A, Igarashi M, Kobayashi M (1997). Natural history and risk factors for immunoglobulin A nephropathy in Japan. Research group on progressive renal diseases. Am J Kidney Dis.

[4] Donadio JV, Grade JP. IgA nephropathy. N Engl J Med. 2002;347:738–48. 10.1056/NEJMra020109 12213946

[5] Strippoli GF, Manno C, Schena FP (2003). An “evidence-based” survey of therapeutic options for IgA nephropathy: assessment and criticism. Am J Kidney Dis.

[6] Samuels JA, Strippoli GF, Craig JC, Schena FP, Molony DA. Cochrane Database Syst Rev. 2003;CD003965. 10.1002/14651858.CD003965 14584001

[7] Xie Y, Nishi S, Ueno M, Imai N, Sakatsume M, Narita I (2003). The efficacy of tonsillectomy on long-term renal survival in patients with IgA nephropathy. Kidney Int.

[8] Pozzi C, Bolasco PG, Fogazzi GB, Andulli S, Altieri P, Ponticelli C (1999). Corticosteroids in IgA nephropathy. A randomized controlled trial. Lancet.

[9] Pozzi C, Andrulli S, Del Vecchio L, Melis P, Fogazzi GB, Altieri P (2004). Corticosteroid effectiveness in IgA nephropathy. Long-term results of a randomized, controlled trial. J Am Soc Nephrol.

[10] Special Study Group (IgA Nephropathy) on Progressive Glomerular Disease. Clinical guideline for immunoglobulin A (IgA) nephropathy in Japan, 3rd version. Jpn J Nephrol. 2011;53(2):123–35.

[11] Kobayashi Y, Fujii K, Hiki Y, Tateno S (1986). Steroid therapy in IgA nephropathy: a prospective pilot study in moderate proteinuric cases. Q J Med.

[12] Hotta O, Miyazaki M, Furuta T, Tomioka S, Chiba S, Horigome I (2001). Tonsillectomy and steroid pulse therapy significanctly impact on clinical remission in patients with IgA nephropathy. Am J Kidney Dis.

[13] Akagi H, Fukushima K, Kosaka M, Doi A, Okano M, Kariya S, et al. A 10-year retrospective case–control study for IgA nephropathy after tonsillectomy. Int Congr Ser. 2003;1257:147–50.

[14] Katafuchi R, Ninomiya T, Mizumasa T, Ikeda K, Kumagai H, Nagata M (2008). The improvement of renal survival with steroid pulse therapy in IgA nephropathy. Nephrol Dial Transplant.

